# Shape analysis of railway ballast stones: curvature-based calculation of particle angularity

**DOI:** 10.1038/s41598-020-62827-w

**Published:** 2020-04-08

**Authors:** Bettina Suhr, William A. Skipper, Roger Lewis, Klaus Six

**Affiliations:** 1 Virtual Vehicle Research GmbH, Rail Systems, Inffeldgasse 21/A, A-8010 Graz, Austria; 2 The University of Sheffield, Department of Mechanical Engineering, Mappin Street, S1 3JD, Sheffield, UK

**Keywords:** Civil engineering, Applied mathematics

## Abstract

Particle shape analysis is conducted, to compare two types of railway ballast: Calcite and Kieselkalk. Focus lies on the characterisation of particle angularity using 3D scanner data. In the literature, angularity is often characterised using 2D data, as these types of data are easier to collect. 3D scanner data contain a vast amount of information (e.g. curvatures) which can be used for shape analysis and angularity characterisation. Literature approaches that use 3D data are often not thoroughly tested, due to a lack of test cases. In this work, two new curvature-based angularity indices are introduced and compared to one from the literature. Analytical test bodies with shapes ranging from spherical towards cubic are used for a first plausibility test. Then, 3D scans of ballast stones are compared to artificially rounded meshes. Only one out of three evaluated angularity indices seem to be suited to characterise angularity correctly in all of the above tests: the newly introduced scaled Willmore energy. A complete shape analysis of the scanned ballast stones is conducted and no difference between the two types of ballast can be seen regarding form, angularity, roughness, sphericity or convexity index. These findings of shape analysis are set in the context of previous works, where experimental results and DEM simulations of uniaxial compression tests and direct shear tests were presented for the same ballast types.

## Introduction

### Shape analysis in the literature

The shape of particles forming a granular material strongly influence its bulk behaviour. To be able to model particle shape in simulations using the Discrete Element Method (DEM), shape analysis can yield helpful insights. In recent years, particle shape analysis has strongly benefited from advances in measurement techniques, considerably increasing the amount of information available. Traditional measurement techniques such as callipers, sieve analysis or photography are now complemented by 2.5D and 3D measurements, see for example^1^, opening up new ways to analyse shape. Particle shape is usually investigated on three different scales: *form*, *angularity*/*roundness* and *texture*. Additionally, *overall shape parameters* exist, which correlate to more than one of the above scales.

To characterise *particle form*, so-called 1D form factors are used. For their calculation the particle’s longest, intermediate and shortest axes ($L,I$, and $S$ respectively) are sought, see^2^ for different definitions and measurement/calculation approaches. From this information, several form factors can be calculated, see^3^ for an overview. Widely used factors include elongation $e=I/L$ and flatness $f=S/I$. Many of the other form factors correlate with elongation or flatness, see^2^.

*Angularity and roundness* are two different concepts to investigate particle shape. The most acknowledged definition of roundness was proposed by Wadell^4–6^, but also other shape descriptors for roundness exist, see^3^ for 2D data or^7–11^ for 3D data. In all aforementioned approaches, only convex areas of the particle are considered for roundness computation. If a particle has concave parts, including edges and corners, then these can be expected to increase particle interlocking, which influences the bulk behaviour of granular materials (e.g. in a direct shear test). As the particles considered in this work show pronounced corners and edges in concave areas, roundness is not considered as an advantageous shape descriptor for these materials.

Therefore, *angularity* will be investigated in this work, as it considers all corners and edges of a particle. Approaches to characterise angularity from 2D data, i.e. images, can be found in^12–14^ or^15^, where several methods are compared. Different types of 3D measurements were used in^12,16,17^ and^18^. The developed angularity index in^18^ includes the integrated mean curvature and is similar to the one introduced later in this work. In^19^ an angularity index was introduced, where the area of the particle’s edges and corners is divided by the total particle surface area. This index will be considered later for comparison.

*Texture* is traditionally analysed from black/white or gray-scale pictures, i.e. 2D data, see^15^ for a comparison of different approaches. In^17^, a 3D surface texture index for 3D voxel meshes was developed .

In the literature, some *overall shape parameters* exist. Sphericity is a frequently used shape parameter and in the literature it was sometimes used to describe particle form. As stated in^8^, this is misleading, as sphericity can be influenced not only by form, but also by roundness/angularity or roughness. The same can be said about ellipseness^20^, or ellipsoidness^21^. Different approaches to characterise shape can be found in the literature, such as a Fourier-based approach^22^, an approach using inertia tensor and inertia moments^23^, an approach using Proper Orthogonal Decomposition^24^, an approach where a flakiness index was defined for railway ballast^25^, and an approach where two convexity indices were introduced^26^.

In this work, *two curvature-based angularity indices* will be introduced and compared to one angularity index from the literature. All three angularity indices use mean curvature values calculated from 3D meshes (generated from 3D scan data). Firstly, the plausibility of the three indices will be checked on analytical test bodies evolving in shape from a sphere towards a cube; the indices methods of operation will be analysed with curvature histograms. Secondly, 3D scans of two types of railway ballast will be used for the evaluation of the three angularity indices. In a final plausibility check, the angularity indices will be tested to see if they can distinguish between the meshes of angular ballast stones and the meshes of rounded stones. This paper will conclude with a complete shape analysis of the scanned ballast stones, i.e. evaluation of form, roughness, and overall shape factors from literature and a correlation analysis.

### The broader picture

The choice of material being considered is motivated by the authors’ previous work. In^27^, the same two types of railway ballast were investigated in uniaxial compression and direct shear tests. While experimental results of the direct shear test were very similar, clear differences could be seen in the uniaxial compression tests (data available^28^). DEM simulations presented in^27^ used the same simple particle shapes (clumps of three non-overlapping spheres) for both types of ballast. While it was not possible to parametrise the simplified Hertz Mindlin contact law such that the simulation results of both compression and direct shear tests agreed well with the experimental measurements, this was successfully done for the Conical Damage contact law. The Conical Damage contact law was originally introduced in^29^ and a detailed description of a slight modification can be found in^30^.

This work can help to answer important questions, that arise from^27^. Do differences in particle shape contribute to the differences in the materials’ bulk behaviour? Are these differences mainly caused by differences in the material properties? Is it reasonable to model both types of ballast with the same particle shape (as done in^27^)? Texture, and respectively roughness information, cannot be modelled in DEM via particle shape, but will be considered in the contact law applied.

Future work will include measurements of material properties for both types of ballast, i.e. Young’s modulus and particle-particle coefficient of friction. There is/will be freely available datasets with shape analysis^31^, material parameters, measured principal experiments^28^ (uniaxial compression tests and direct shear test) for both types of railway ballast. This data set, which will allow analysis of the material bulk behaviour, regarding particle shape and material properties, as well as providing all relevant information to develop DEM model parametrisation and validation strategies.

## 3D scan data

### Railway ballast

In this work, two different types of railway ballast “Calcite” (stems from Croatia) and “Kieselkalk”, also known as Helvetic Siliceous Limestone, (stems from Switzerland) was considered. Please note that the Calcite ballast does not consist of the mineral calcite: CaCO${}_{3}$. These ballast types are two out of five types tested at Graz University of Technology at the Institute of Railway Engineering and Transport Economy in the project “LoadLabs” (project partners: Deutsche Bahn (DB), Austrian Federal Railways (ÖBB), Swiss Federal Railways (SBB) and Schweizerische Südostbahn (SOB), Institute of Railway Engineering and Transport Economy: Graz University of Technology), see^32^ (in German, abstract available in English). In a previous paper^27^, compression tests as well as direct shear tests for these two types of ballast were conducted^28^.

In this paper, 3D scans of single stones of both types of ballast were taken (data available^31^). The scans were taken using the Fusion FAROARM^®^ with an attached laser scan arm, the resolution of the scans can be considered to be 0.01mm. The scanning method is as follows: a stone was fixed onto a scanning stage and the upper part of the stone was scanned. Then, the stone was turned over with the upper end then being fixed thus allowing the lower part to be scanned. Each of these two scans yielded a point cloud of a part of the stones surface. Due to local irregularities sometimes holes occurred in these point clouds. Both parts of the stone’s surface were aligned using the global registration algorithm built into the Geomagic studio software, and a triangular mesh of the stone’s surface was generated. The scanning and mesh generation process took about 1h per stone. The generated meshes were cleaned in an automated process using the open-source software MeshLab^33^.

**Figure 1:**
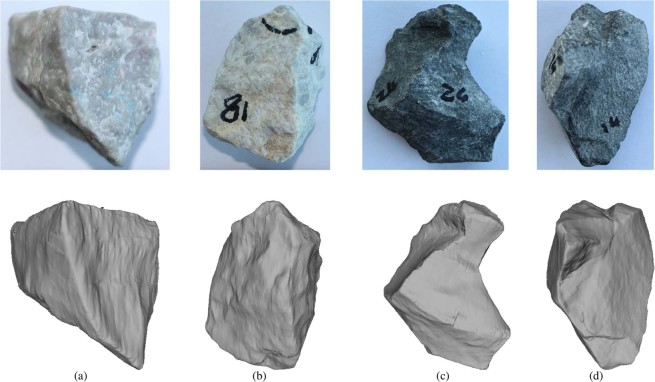
Examples of ballast stones together with meshes of their scanned versions: (**a**) and (**b**) Calcite, (**c**) and (**d**) Kieselkalk. To give an impression of the stones’s size, the longest axis, $L$ of their bounding box is given: (**a**) $L=32$ mm, (**b**) $L=46$ mm, (**c**) $L=56$ mm, (**d**) $L=51$ mm.

Some examples of Calcite and Kieselkalk stones together with meshes of their scanned versions can be seen in Fig. 1. The sieve size curves of both types of ballast can be found in^27^. The maximal sieve size was 64 mm. The sizes of the bounding boxes of each scanned stones can be found in the data set^31^. For each type of ballast, 25 stones were scanned. The stones were chosen by one of the authors randomly. In the conclusion section, it will be addressed if the number of scanned stones is high enough to support the drawn conclusions.

### Artificially rounded ballast

**Figure 2:**
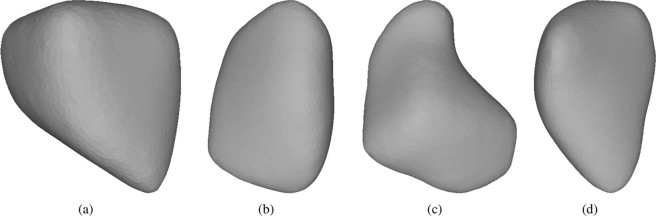
Examples of artificially rounded meshes: (**a**) and (**b**) Calcite, (**c**) and (**d**) Kieselkalk. Compare with Fig. 1 for original scanned meshes.

As previously described, this paper deals with the characterisation of particle angularity. To be able to test methods developed later in this paper, it is advantageous to compare the angular ballast stones to relatively round stones, such as river pebbles. As such data is not available, rounded objects are created for test purposes by smoothing and simplifying the scanned meshes. Several available methods from the the open-source software MeshLab^33^ were combined. To allow comparison between the original stones and the artificially rounded ones, the same stones as in Fig. 1 are shown in Fig. 2 in their rounded versions.

## Characterisation of angularity

### Angularity index definition

When characterising angularity or roundness, it is common in the literature to calculate single value angularity indices. While it is convenient to reduce all information from a 3D mesh to a single number, it is even more important that this number makes best use of the available information. In^19^, the curvature at each mesh vertex is calculated to identify corners. Then, the ratio between the surface area associated with these corners and the total surface area is defined as the angularity index, thus the calculated curvature values are not directly used.

This work aims to introduce a curvature-based angularity index which: makes best use of the available information from the 3D mesh, assigns high values to angular particles and low values to round particles, and is easy to calculate. Throughout this work the mean curvature, $H$, will be used, which is the mean value of the two principal curvatures, ${k}_{1},{k}_{2}$. At a point on a surface, these principal curvatures can be understood as the maximum and minimum values of curvature of the intersection of the surface with all normal planes on this point (i.e. curvatures of plane curves). To construct an angularity index, two integrals over curvature values will be compared: the total curvature, ${t}_{c}$, and the Willmore-energy, ${W}_{e}$. The particle surface area is denoted by $A$ and the mean curvature by $H$. Then, total curvature and Willmore energy are defined as:

1 $${t}_{c}={\int }_{A}H\,da$$

2 $${W}_{e}={\int }_{A}{H}^{2}da$$

The unit of the total curvature is metres. Total curvature is similar to the inverse of the shape parameter introduced in^18^, where the curvature is calculated from the particle surface reconstructed by spherical harmonic series. The use of spherical harmonic series for particle shape analysis has the advantage of being able to distinguish between different multi-scale elements of shape: form, angularity and texture^12^. However, it demands a large amount of computer memory and computational time. Therefore, the approach presented here, which is easier to calculate, is chosen. In differential geometry, the Willmore energy is a measure of the bending energy of a surface and it is dimensionless.

Both total curvature and Willmore energy can be easily calculated with the provided information by state-of-the-art software tools. In this case, the open-source software PyMesh^34^ is used. At each vertex of the mesh, ${v}_{i}$, the vertex area is denoted by ${A}_{i}$ and the mean curvature at the vertex by ${H}_{i}$. As the curvature is constant over the vertex area, the integrals in Eqs. (1) and (2) reduce to simple expressions:

3 $${t}_{c}={\sum }_{i=1}^{N}{A}_{i}\ {H}_{i}$$

4 $${W}_{e}={\sum }_{i=1}^{N}{A}_{i}\ {H}_{i}^{2}$$

Both total curvature and Willmore energy can be scaled, such that they are dimensionless and equal 1 for a sphere. Thus, the following scaling is introduced, where ${r}_{eq}$ denotes the radius of the surface equivalent sphere.

5 $$\widehat{{t}_{c}}=\frac{1}{4\pi {r}_{eq}}{\int }_{A}H\,da$$

6 $${\widehat{W}}_{e}=\frac{1}{4\pi }{\int }_{A}{H}^{2}da$$

For comparison, the angularity index ${I}_{A}$, introduced in^19^, will also be considered in this work. For its calculation, the radius of the largest inscribed sphere, ${r}_{inscr}$, has to be calculated. Also, a modified mean curvature, $\widetilde{H}$, is used for its calculation. All elements of the mesh with a higher curvature than the inscribed sphere are regarded as edges or corners. ${I}_{A}$ is calculated as follows:

7 $$\widetilde{H}=\frac{| {k}_{1}| +| {k}_{2}| }{2}$$

8 $${I}_{A}=\frac{{\sum }_{i=1}^{N}{A}_{i}\ \max \left(0,\,{\rm{sign}}\,\left(\widetilde{{H}_{i}}-\frac{1}{{r}_{inscr}}\right)\right)}{A}$$

Thus, the sum of the surface area of these edges and corners is divided by the total area, which gives the angularity index ${I}_{A}$. To use the largest inscribed sphere’s radius for edge and corner detection is convenient as it avoids introducing a threshold for curvatures. However, in this approach particle shape influences the angularity. The computational effort for this index is higher than for the two other indices, as the largest inscribed sphere must be calculated first. The ${I}_{A}$ index assigns 0 to a sphere and higher values for more angular particles, where the maximal value is 1. An example of an artificial shape with ${I}_{A}=1$ is given in^19^ as a starlike shape, which is composed of many spikes, while having a relatively large value of ${r}_{inscr}$.

### Plausibility check: analytical test bodies

To check the ability of the introduced angularity indices to measure particle angularity, test bodies will be introduced. These test bodies have a shape parameter, $p$, and evolve from a sphere towards a cube. They are obtained by transforming the coordinates, $x$, of a unit sphere to hold:

9 $$| | x| {| }_{p}={\left(\mathop{\sum }\limits_{k=1}^{3}{x}_{k}^{p}\right)}^{\frac{1}{p}}=1$$

**Figure 3:**
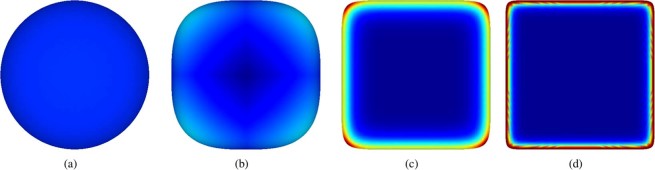
Test bodies with shape parameter, $p$, coloured by mean curvature in the range of 0 to 5: (**a**) sphere: $p=2$, (**b**) $p=3$, (**c**) $p=9$, (**d**) $p=20$.

$p=2$ describes a sphere. With increasing $p$ the test body tends towards a cube, as can be seen in Fig. 3 (these test bodies are “unit spheres” in the ${L}^{p}$ space).

**Figure 4:**
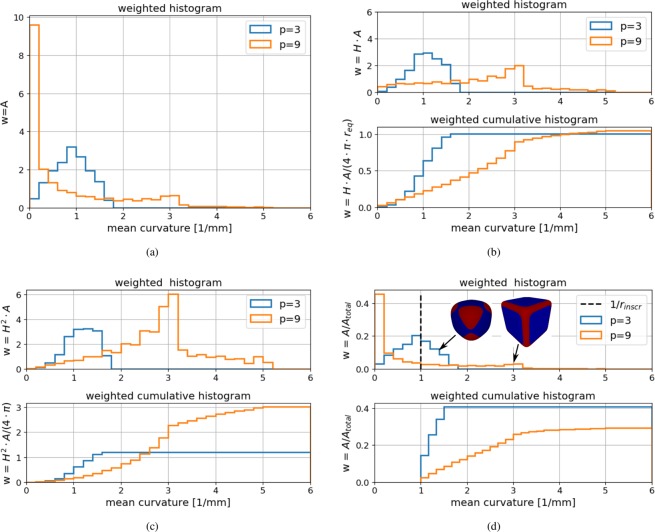
Weighted histograms illustrating the different angularity indices for two test bodies: (**a**) mean curvature $H$: histogram weighted by vertex area, (**b**) scaled total curvature: $\widehat{{t}_{c}}$, (**c**) scaled Willmore energy: ${\widehat{W}}_{e}$, (**d**) ${I}_{A}$ angularity index from^19^.

With increasing $p$, the angularity of the test bodies increases, which should be reflected by an angularity index. To visualise how the three different angularity indices work, Fig. 4a shows a histogram of the mean curvature for both a rounded test body (p = 3) and a more angular one (p = 9). In this histogram, the mean curvature values are weighted by the corresponding vertex area. The curvature values of the rounded test body (p = 3) are distributed between 0 and 2 and the biggest area of this body has a curvature of 1. In contrast, the more angular test body with $p=9$ has a high amount of (nearly) plane areas with curvatures nearly 0 and corners and edges are composed of curvature values up to 3.

The calculation of the scaled total curvature is analysed in the weighted histograms in Fig. 4b. In the upper plot, the used weights are the curvature multiplied by the vertex area, i.e. the summand of Eq. (3). In the lower subplot, the cumulative histogram is additionally weighted with the scaling factors of the scaled total curvature. This histogram visualises a discretisation of the integration in Eq. (5), with its final values equal to the values of the scaled total curvature for the two test bodies. From this plot it can be seen which curvature values contribute most to the calculation of $\widehat{{t}_{c}}$. For the rounded test body, $p=3$, the low curvatures (below 1) contribute almost half to the total values of $\widehat{{t}_{c}}$. In contrast, for the more angular test body, $p=9$, the main contribution to  $\widehat{{t}_{c}}$ comes from the higher curvatures. Nevertheless, both test bodies are assigned a similar value just slightly above 1, because the loss of contributions at a certain curvature is nearly compensated by the increase at other curvatures.

Figure 4c shows the analogue plots for the scaled Willmore energy. In the upper subplot, the weight ${H}^{2}\cdot A$ is used and in the lower subplot the cumulative histogram is weighted by ${H}^{2}\cdot A/(4\cdot \pi )$. Again, the integral in Eq. (6) is visualised, so that the final value of the cumulative histogram equals the value of ${\widehat{W}}_{e}$. Curvature values below one are weighted less by the quadratic scaling in Fig. 4c and values above one are weighted more, when compared to the linear scaling in Fig. 4b. Therefore, nearly flat parts of the particle surface contribute very little to ${\widehat{W}}_{e}$, while the curvatures of corners and edges are weighted more. The resulting values of the scaled Willmore energy reflect well the difference in angularity between both test bodies.

In the upper part of Fig. 4d, the weighted histogram for the visualisation of ${I}_{A}$ is shown. The weights used are the vertex area divided by the total surface area of the particle. The curvature of the largest inscribed sphere, $1/{r}_{inscr}=1$, is also shown as a dashed line. Additionally, both test bodies are shown with areas of curvature higher than $1/{r}_{inscr}$ coloured red. For the calculation of ${I}_{A}$, all vertex areas belonging to curvatures bigger than $1/{r}_{inscr}$ are summed up and divided by the total surface area. This is shown in the lower subplot, where the weighted cumulative histogram of all curvatures greater than 1 is shown. This angularity index makes no difference between the area of the rounded test body, which has a curvature slightly above 1, and the corners and edges of the angular test body. As a consequence, the more rounded test body is assigned a higher angularity index than the angular test body: ${I}_{A}(p=3)=0.4$ and ${I}_{A}(p=9)=0.3$.

**Figure 5:**
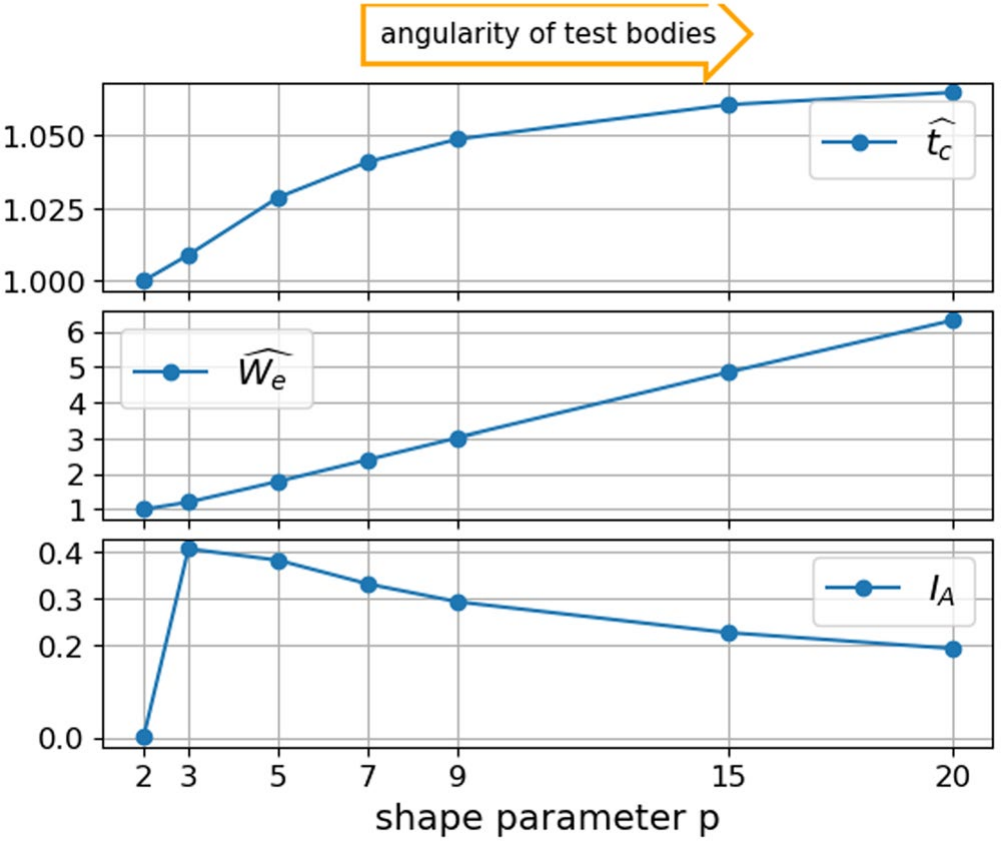
Comparison of scaled total curvature, scaled Willmore energy and angularity index ${I}_{A}$ from^19^ on test bodies.

After discussing the mode of operation of the three different angularity indices, they will now be evaluated for all test bodies, as shown in Fig. 5. Both the scaled total curvature and the scaled Willmore energy assign 1 to a sphere and higher values to the more angular test bodies as shape parameter $p$ increases. The scaled total curvature assigns 1 to a sphere and 1.065 to the most angular test body with $p=20$. This narrow range of values makes the results hard to interpret. In contrast, the scaled Willmore energy of the $p=20$ test body is 6.3. For the authors, this naturally wider range of the scaled Willmore energy does fit better to the intuitively perceived angularity of the test bodies. In the third subplot of Fig. 5 the ${I}_{A}$ index is shown. By construction, it assigns 0 to the sphere ($p=2$). Then, the quite rounded test body, $p=3$, is assigned the highest ${I}_{A}$ value. With increasing angularity of the test bodies the ${I}_{A}$ value deceases. The reason for this behaviour is that the ${I}_{A}$ index does not make use of the modified mean curvature itself but only uses it to identify the area of edges and corners. For $p=20$ the edges and corners have a small area but a high curvature; this is not reflected by the ${I}_{A}$ index. The area of edges and corners is part of the roundness characterisation used in^9,10^ and^8^, but here local curvatures or locally fitted spheres are considered directly for roundness computation. Therefore, the roundness definition developed in^10^ successfully classifies the angularity of analytical test bodies. The behaviour seen from the ${I}_{A}$ index so far is opposite to the expected behaviour of an angularity index.

### Scanned meshes: pre-processing and artificial rounding

**Figure 6:**
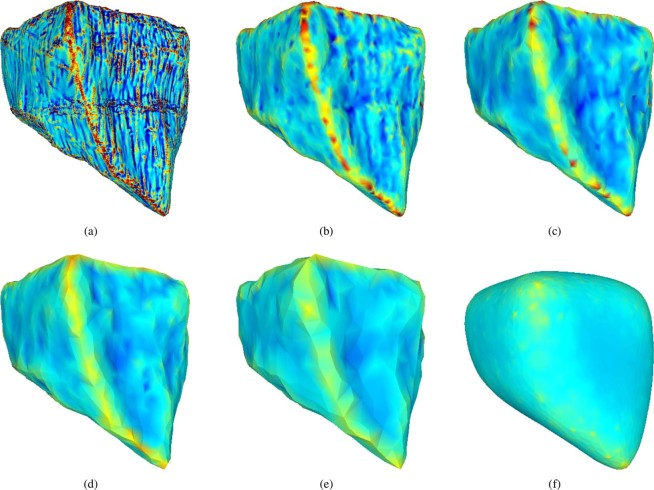
Example of mesh simplification levels and artificially rounded meshes. Meshes coloured by mean curvature in the range of $-0.5$ and 1: (**a**) cleaned mesh, (**b**) CSE1, (**c**) CSE2, (**d**) CSE3, (**e**) CSE4, (**f**) rounded mesh.

In this section, the angularity of the two types of ballast will be investigated and compared to the artificially rounded meshes. For this purpose, the 3D scans presented in the previous Section will be used (25 scans for each type of ballast). Due to the high spatial resolution of the scans, information of the stones’ texture and roughness is also included in the resulting triangular meshes. As the angularity of the stones will be characterised via the local curvatures of the stones, it is advisable to remove roughness/texture information from the meshes,^8,10,19^. In^10^, a feature preserving smoothing algorithm is applied. Their meshes show a noise-like type of roughness, which is successfully removed by the smoothing algorithm, while preserving sharp edges and corners. It is important to keep in mind that the chosen smoothing method and the amount of smoothing will strongly influence the curvatures computed later. In^11^, isolated high-curvature features are excluded from roundness computation by considering high local curvature together with a large relative connected area. In this paper the scanned stones show a more structured texture with sharp rills, which should be removed, see Fig. 6a for an example stone. As the rills are partially connected, the method proposed in^11^ could not be applied. Also, the authors could not achieve removal of the texture via smoothing, although several smoothing algorithms available in MeshLab^33^ were applied. In^8^ and^19^ roughness/texture of the meshes is removed by reduction of the number of vertices/facets forming the mesh. As stated in^8^, the amount of reduction (number of facets in the reduced mesh) massively influences the computed curvatures. Moreover, the chosen method for mesh simplification can be expected to influence the results as well.

In this work, the “collapse_short_edges” algorithm from PyMesh^34^ will be used for mesh simplification, denoted as CSE. The CSE algorithm tries to collapse all edges of a given mesh that are shorter than a given threshold, and aims to be feature preserving. From the simplified meshes the mean curvature is calculated, allowing the evaluation of the three angularity indices. The question of how much a mesh should be simplified will be discussed next.

**Figure 7:**
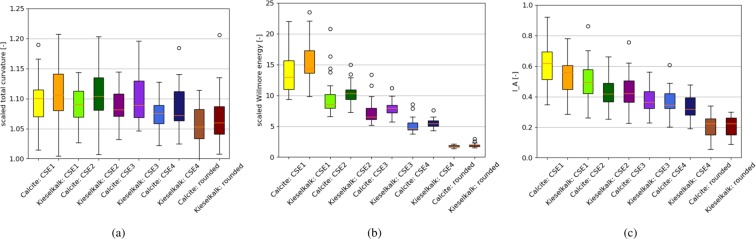
Comparison of different degrees of mesh simplification and artificially rounded meshes for different angularity indices: (**a**) scaled total curvature: $\widehat{{t}_{c}}$, (**b**) scaled Willmore energy: ${\widehat{W}}_{e}$, (**c**) ${I}_{A}$ angularity index from^19^.

Four levels of mesh simplification will be considered in this work, denoted by CSE1 to CSE4. As the CSE algorithm cannot reduce meshes to a given number of elements, the number of elements in the simplified meshes will vary between different meshes; in Table 1 the median values are given. In the literature,^8^ and^19^ use meshes reduced to 1500 elements for their angularity calculation, which would roughly correspond to the CSE4 level. Figure 6 shows for one example stone: the scanned (cleaned) mesh and the four different mesh simplification levels. All meshes are coloured by the mean curvature and the aforementioned rilled texture is increasingly removed as the mesh is simplified. As an additional test for the angularity indices, the angular meshes will be compared to the artificially rounded meshes. The median number of elements for these artificially rounded meshes are given in Table 1 and for comparison, the artificially rounded mesh is also shown in Fig. 6. Figure 7 shows the computed angularity indices for all scanned meshes at different levels of mesh simplification and the artificially rounded meshes. To visualise the calculated angularity values for each group of meshes, boxplots are used. Here, the median value is shown with a line, the central 50% of the data is enclosed in the box (values between the 25th and 75th percentile). The so-called whiskers extend to (the last datum below/above) 1.5 times the spread of this central 50% (interquartile range). Values outside of the whiskers are considered outliers and are plotted with separate markers. In Fig. 7a, the scaled total curvature is shown for the meshes of Calcite and Kieselkalk stones: their different levels of simplification, and the artificially rounded meshes. In the computed $\widehat{{t}_{c}}$ values, little influence of the mesh simplification is seen, i.e. its range of values overlaps strongly for the least and most simplified meshes. Moreover, the artificially rounded meshes are assigned similar/same values as the angular meshes. In Table 2, $\widehat{{t}_{c}}$ values of the angular (CSE4 simplified) and rounded meshes shown in Figs. 1 and 2 are given. The rounded meshes 2c and 2d are assigned higher angularity values than the angular meshes 1a and 1b. Therefore, the scaled total curvature is considered a bad indicator of particle angularity.

**Table 1:** Median number of elements of: the meshes using four different levels of mesh simplification and the artificially rounded meshes.

	CSE1	CSE2	CSE3	CSE4	Rounded
Calcite	16600	7300	4000	1800	6800
Kieselkalk	19600	8700	4800	2100	7700

**Table 2:** Comparison of angularity indices evaluated for angular meshes (CSE4 simplified) shown in Fig. 1 and rounded meshes shown in Fig. 2.

angular	$\widehat{{{\boldsymbol{t}}}_{{\boldsymbol{c}}}}$	rounded	$\widehat{{{\boldsymbol{t}}}_{{\boldsymbol{c}}}}$	angular	${\widehat{{\boldsymbol{W}}}}_{{\boldsymbol{e}}}$	rounded	${\widehat{{\boldsymbol{W}}}}_{{\boldsymbol{e}}}$	angular	${{\boldsymbol{I}}}_{{\boldsymbol{A}}}$	rounded	${{\boldsymbol{I}}}_{{\boldsymbol{A}}}$
1a	1.04	2a	1.00	1a	4.36	2a	1.57	1a	0.42	2a	0.34
1b	1.05	2b	1.03	1b	4.99	2b	1.74	1b	0.49	2b	0.26
1c	1.14	2c	1.13	1c	6.31	2c	2.55	1c	0.30	2c	0.21
1d	1.07	2d	1.06	1d	5.33	2d	1.87	1d	0.31	2d	0.21

The scaled Willmore energy is plotted in Fig. 7b (for better visualisation three outliers of Calcite CSE1 between 25 and 35 are not shown). A strong decrease in the scaled Willmore energy can be seen with increasing mesh simplification (i.e. decreasing number of elements). This means that the meshes’ roughness/texture, which should be removed by the mesh simplification, has a big influence on the obtained results. For all levels of mesh simplification, the median of the calculated values is higher for Kieselkalk than for Calcite. However, the range of values overlaps nearly completely for both types of ballast for all levels of mesh simplification. Thus, if the scaled Willmore energy is used to characterise particle angularity, no difference can be seen between Calcite and Kieselkalk ballast stones. Comparing the angular and the rounded meshes, the calculated values are separated completely. This can also be seen in Table 2. With the results obtained so far, the scaled Willmore energy seems to be well suited for use as an angularity index.

The angularity index, ${I}_{A}$, is evaluated as well and shown in Fig. 7c. It can be seen that increasing mesh simplification reduces the calculated ${I}_{A}$ index. The same dependency on the meshes’ roughness/texture that was seen with the scaled Willmore energy can be observed, but to a reduced extent. The ${I}_{A}$ index characterises Calcite as slightly more angular than Kiesekalk. Both the scaled Willmore energy and the ${I}_{A}$ index give very similar results for Calcite and Kieselkalk for all levels of mesh simplification. Therefore, it can be concluded that both types of ballast have similar angularity. A drawback of the ${I}_{A}$ index is seen when angular meshes are compared to the artificially rounded meshes; the range of the computed values overlaps clearly. An example in given in Table 2, where the rounded mesh 2a is assigned higher angularity than the angular meshes 1c and 1d. The ${I}_{A}$ index uses the modified mean curvature only to identify areas of edges and corners, but not for the calculation of ${I}_{A}$ itself. Therefore, a clear separation between rounded and angular meshes fails. For very rounded particles, e.g. river pebbles, the ${I}_{A}$ index may better separate angular and round particles. Summarising, the ${I}_{A}$ index had problems with classifying the angularity of analytical test bodies, as well as angular and rounded meshes. Thus, it is not considered a suitable angularity index in this work.

**Figure 8:**
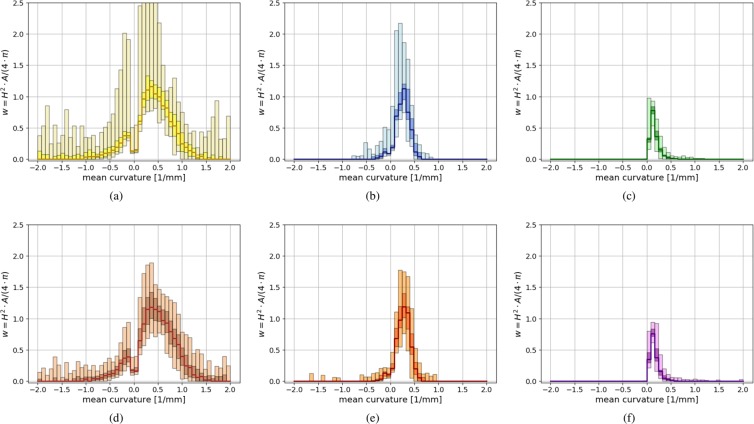
Comparison of CSE1 and CSE4 simplified angular meshes to artificially rounded meshes for Calcite and Kieselkalk scans. Shown is a summary of weighted curvature histograms, where weights correspond to ${\widehat{W}}_{e}$: (**a**) CSE1 Calcite, (**b**) CSE4 Calcite, (**c**) rounded Calcite, (**d**) CSE1 Kieselkalk, (**e**) CSE4 Kieselkalk, (**f**) rounded Kieselkalk.

From the analysis presented above, it is hard to conclude which is the optimal level of mesh simplification. The level CSE4 is close to the amount of simplification found in the literature, see^8^ and^19^. For a more detailed understanding of the mode of operation of the ${\widehat{W}}_{e}$ angularity index, Fig. 8 shows similar information for the CSE1 simplified, CSE4 simplified and artificially rounded meshes than Fig. 4 shows for the test bodies. Weighted histograms are shown, which contain the information for all 25 scanned meshes of one type of railway ballast. To visualise the scattering between the histograms of one type of railway ballast the following plotting scheme is chosen. In every bin 25 values exist (belonging to the 25 scans per ballast type). The median of these values is shown as a line-plot. The range covered by the central 50% is shown with a dark shaded rectangle, while the minimum/maximum of all values is shown with a light shaded box. This representation loosely connects a histogram and a box plot. Due to the used weights, it can be seen how much each curvature bin contributes to the scaled Willmore energy.

Figure 8a,d show the results for CSE1 simplified meshes for Calcite and Kieselkalk, respectively. The histograms scatter quite a lot for both types of ballast. For Calcite some curvature values reach up to 3.5. In Fig. 8a these results are cut for improved readability. As the curvature values are squared in the weights, negative curvatures increase the angularity value. For both Calcite and Kieselkalk the main contributions in the calculation of ${\widehat{W}}_{e}$ stem from curvatures around 0.5. As described before: the quadratic weighting of the curvature maps reduces the contribution of nearly flat areas and increases the contribution of edges and corners with a higher curvature. This also explains the dip at the bins with nearly zero curvature.

Figure 8b,e show the analogue plots for CSE4 mesh simplification level for Calcite and Kieselkalk respectively. The scattering of the results is reduced by the mesh simplification. The contributions from negative curvatures is reduced considerably for both types of ballast. Moreover, the main contributions in the calculation of ${\widehat{W}}_{e}$ stem from curvatures around 0.25, where curvatures above 0.5 are negligible. The CSE1 meshes still contain texture information, which is almost completely removed in the CSE4 meshes. During the simplification, the curvature at edges and corners is reduced, thus the main contributions in the calculation of ${\widehat{W}}_{e}$ move closer towards zero (for both negative and positive curvatures). The loss in texture information corresponds to a more narrower of curvatures occurring.

The corresponding plots for the artificially rounded meshes are shown in Fig. 8c,f. In this representation, several differences can be seen between angular and rounded meshes. The rounded meshes show nearly no contribution from negative curvatures. Negative mean curvature is related to either concave areas or saddle points. These areas are thus almost completely removed by the applied simplification and smoothing methods. The rounded meshes clearly show less scattering than the angular meshes. Their main contributions to the calculation of ${\widehat{W}}_{e}$ stem from curvatures around 0.1 while values above 0.25 contribute very little. This shows that the curvature of edges and corners was successfully reduced by the rounding procedure.

This analysis confirms that the scaled Willomore energy seems to be well suited to characterise angularity.

As previously seen in Figs. 7 and 8 shows that no distinct differences can be seen in the angularity of Calcite and Kieselkalk.

## Evaluation of classical shape descriptors

In this section, both types of railway ballast will be compared using classical shape factors for describing form, roughness and mixed quantities like the sphericity and the convexity index.

### Form

**Figure 9:**
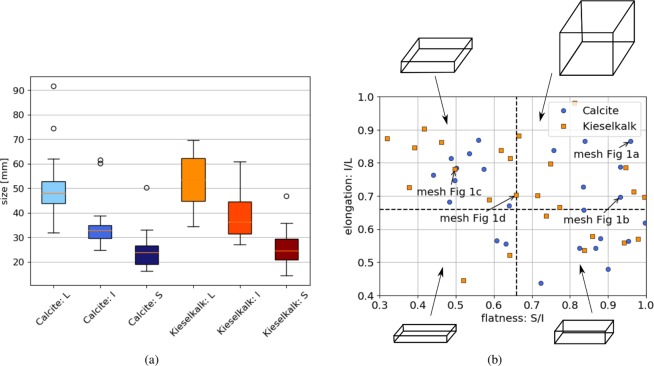
Comparison of particle form and size: (**a**) boxplot of particle sizes for Calcite and Kieselkalk, (**b**) elongation over flatness.

1D shape descriptors, or form factors, are widely used to characterise particle form. The particle’s longest, intermediate and shortest axes are sought, usually these axes are defined to be perpendicular. While there are several ways to compute these values, see e.g.^2^, here a bounding box of minimum volume is calculated for the scanned meshes, using the Matlab code^35^ (thus the axes are perpendicular). With these three axes, mostly denoted as $L,I,S$, the two most important form factors can be computed: elongation $e=I/L$ and flatness $f=S/I$. Moreover, many other form factors exist that use $L,I,S$, see e.g.^2,3^. In^2^, seven different form factors were evaluated and all of them were found to correlate strongly with either elongation or flatness. Therefore, in this work only $L,I,S$ as well as elongation and flatness, will be evaluated. Figure 9a shows the results of the minimum bounding box calculation for Calcite and Kieselkalk meshes. While the overall size of both types of ballast is similar, the Kieselkalk stones have slightly larger values for $L$ and $I$. In Fig. 9b, elongation is plotted over flatness for both Calcite and Kieselkalk. Regarding flatness and elongation, no distinct differences can be seen in particle form between Calcite and Kieselkalk. The plot also contains dashed lines at 2/3 along the axes to distinguish between the four different classes of shape^3^, visualised with drawn boxes. Less than half of the plotted values lie in the upper right corner, denoted by Zingg as “spherical”. Most stones are classified to be “flat”, i.e. their flatness and elongation values are positioned in the upper left corner of the plot. Only four stones are “flat and columnar” (lower left corner) and the rest are “columnar” (lower right corner).

### Roughness

Roughness values are calculated, in an automated procedure, from the cleaned 3D scans. For each mesh, 642 points are considered for roughness computation. To identify these points, a sphere with 642 vertices (continuously distributed over its surface) is generated and the spherical coordinates of its vertices are calculated. The corresponding polar and azimuth angles are used to identify the closest point of the scanned meshes with the same polar and azimuth angles. Then, the roughness is calculated using all points in a 2.5 mm radius. A plane is fitted through all points within this radius in a least squares sense. The deviation from this plane, ${d}_{i}$, is used to calculate the roughness value, ${S}_{q}$.

10 $${S}_{q}=\sqrt{\frac{1}{N}{\sum }_{i=1}^{N}{d}_{i}^{2}}$$

In this way 642 roughness values are computed for each mesh. In the described algorithm, the underlying geometry of the mesh is not checked. This means that roughness is also calculated at non-planar areas, where the geometry of the stone (e.g. edges and corners) contributes most to the computation of ${S}_{q}$. Therefore, for each stone only the 10 smallest values are considered.

**Figure 10:**
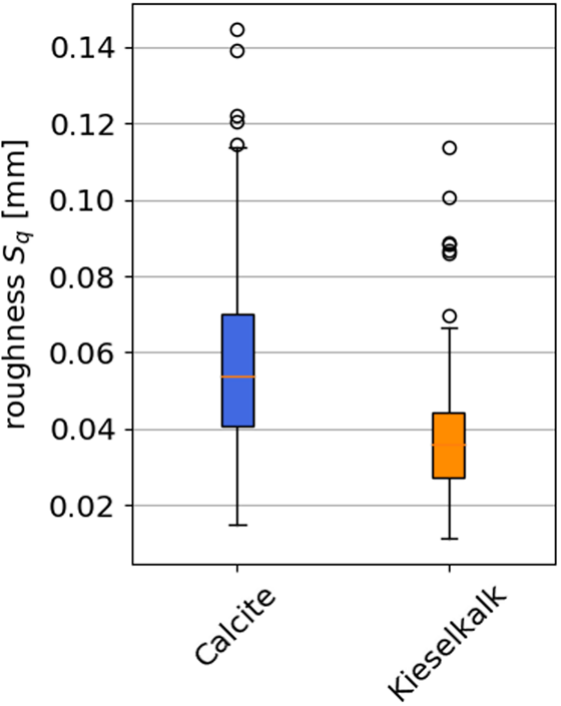
Comparison of roughness ${S}_{q}$ for Calcite and Kieselkalk.

The calculated roughness’ for Calcite and Kieselkalk are shown in Fig. 10. The resulting values for Kieselkalk scatter less than the values for Calcite. Also, Kieselkalk shows smaller roughness values than Calcite, comparing the median of their values. In general, roughness calculation using 3D scanned data is not optimal, because the number of points used for the ${S}_{q}$ calculation varies. From the results obtained, it seems that Kieselkalk has a lower roughness than Calcite. Nevertheless, additional measurements would be needed to ensure this interpretation.

### Overall shape parameters

In this subsection, the particles’ sphericity, $\psi$, and convexity index, $c$, will be evaluated, both of which are frequently used shape descriptors. For the calculation of sphericity, the particle volume, $V$, is used to calculate the surface area of a sphere with equal volume. This value is then divided by the actual particle surface area, $A$.

11 $$\psi =\sqrt[3]{36\pi {V}^{2}}/A$$

Thus, the sphericity is equal to one for a sphere and decreases for less spherical objects. In the literature, sphericity was sometimes used to describe particle form. As stated in^8^, this is misleading, as sphericity can be influenced not only by form but also by roundness/angularity or roughness. If the particle volume is imagined to be fixed, then changes in roundness or roughness will cause changes in the particle surface and thus lead to different a sphericity. Therefore, sphericity is better understood as an overall shape parameter, than a form factor.

The convexity index is defined as the ratio between the volume of the particle’s convex hull and the particle’s volume. The convex hull is the smallest convex shape, which completely encloses the particle. Hence, the convexity index is equal to one for convex particles and greater than one for general shapes.

12 $$c=V(\,{\rm{convex\; hull}}\,)/V(\,{\rm{particle}}\,)$$

**Figure 11:**
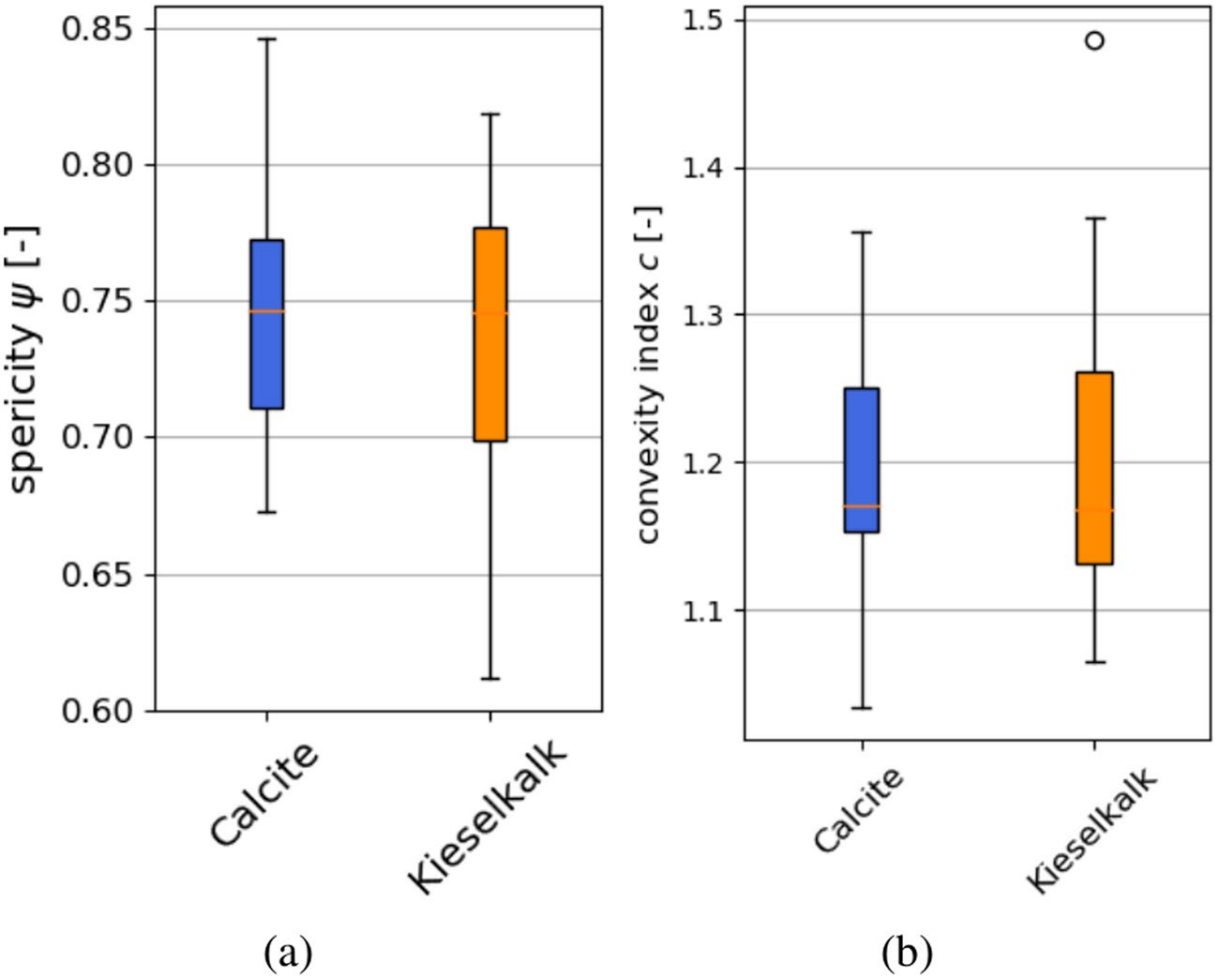
Comparison of particle overall shape parameters: (**a**) sphericity, (**b**) convexity index.

For the calculation of sphericity and the convexity index, particle volume and the volume of the convex hull is calculated by Meshlab,^33^ and particle surface is obtained as the sum of the area of all triangles in the mesh. The convex hull of the particles is calculated via PyMesh^34^. Figure 11 shows the calculated sphericity and convexity index values for Calcite and Kieselkalk meshes. Little difference can be seen between both types of ballast regarding the spericity. From the scanned stones, the Calcite sample contains the most spherical stones with $\psi$ values nearly 0.85, while the Kieselkalk sample contains less spherical stones with $\psi$ nearly 0.6. The median of $\psi$ is almost identical for both types of ballast. Regarding the convexity index, shown in Fig. 11b, both types of ballast show values in the same range and the median values almost coincide. Only one outlier of Kieselkalk is more concave than the other stones considered. To get a visual impression of the calculated $\psi$ and $c$ values, Table 3 gives the values for the meshes shown in Fig. 1. The outlier in the convexity values of Kieselkalk is the mesh in Fig. 1c.

**Table 3:** Evaluated $\psi ,c$ for meshes shown in Fig. 1.

mesh	$\psi$	$c$
1a	0.80	1.19
1b	0.78	1.17
1c	0.64	1.49
1d	0.73	1.21

### Correlation analysis

Particle form, angularity, roughness are investigated separately, as they are seen as different scales and/or dimensions of shape. With a correlation analysis,can check whether the calculated shape descriptors are really independent from each other or not.

**Figure 12:**
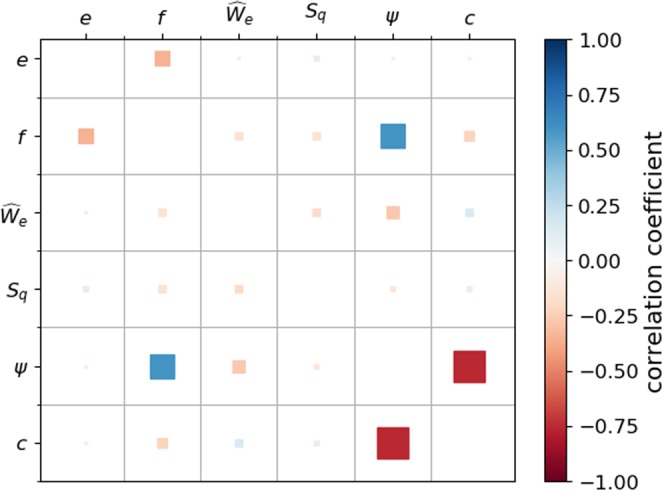
Scatter plot showing correlations between $e$, $f$, ${\widehat{W}}_{e}$, ${S}_{q}$, $\psi$ and $c$.

Considered shape descriptors are: the form factors elongation $e$ and flatness $f$, the scaled Willmore energy ${\widehat{W}}_{e}$ as angularity index, ${S}_{q}$ for roughness, and $\psi$ and $c$ as overall shape parameters. For this analysis, it is necessary to choose one roughness value per stone so the median of the 10 calculated values is taken. Fig. 12 shows a correlation matrix between the evaluated quantities based on Pearson correlation coefficients. For improved readability, the correlation of a quantity with itself is not plotted. Pearson correlation coefficients are sensitive to linear relations between two quantities and range from $-1$ to +1, distinguishing between negative and positive correlation. Non-linear correlations will be missclassified by the Pearson coefficient. An additional visual inspection ensured that this was not the case.

Low correlations can be seen between form $e,f$, angularity ${\widehat{W}}_{e}$ and roughness ${S}_{q}$. Therefore, it can be concluded that these dimensions of shape were successfully separated in the shape analysis. The overall shape parameters $\psi$ and $c$ are correlated to a moderate extent. This correlation was also reported in the literature^8^, indicating that these parameters are not independent from each other and it might be enough to investigate only one of them. Apart from this correlation, only one, between $\psi$ and $f$ is established (to a weak extent). The lack of correlation between $c,\psi$, angularity and roughness show that for the considered particles none of the influences stated in^8^ were present.

## Conclusions

In this paper, two easy to compute, curvature-based angularity indices are introduced. Their performance is compared to the ${I}_{A}$ angularity index from^19^. A first plausibility test is conducted on analytical test bodies ranging in shape from a sphere towards a cube. Then, the angularity of scanned stones made up of two types of ballast, Calcite and Kieselkalk, is evaluated. Finally, a comparison with artificially rounded meshes shows that the newly defined scaled Willmore energy is most suitable index to characterise particle angularity. Regarding Calcite and Kieselkalk, none of the considered angularity indices show a clear difference between the two types of railway ballast.

This paper concludes with a complete shape analysis of the scanned stones for both types of railway ballast. The shape descriptors evaluated are: 1D form factors (i.e. elongation and flatness), angularity, roughness, sphericity and the convexity index. For all these quantities, excluding roughness, Calcite and Kieselkalk yield very similar results due to the strongly overlapping values of the shape descriptors. In this case, the authors consider the number of 25 scans to be sufficient. Even if additional scans were to yield different shape descriptor values for Calcite and Kieselkalk, the 25 scans already measured will still have strongly overlapping values. Thus, additional scans could change the finding of this manuscript in only a limited way. Therefore, no further scans were considered necessary to support our findings: It is concluded that Calcite and Kieselkalk are similar in shape.

The same two types of railway ballast were investigated in^27^, where measurements and DEM simulations of uniaxial compression and direct shear test were presented. The experimental results of the direct shear test were very similar for Calcite and Kieselkalk. In contrast, in the uniaxial compression test clear differences could be seen in the stiffnesses and slight differences could be seen in the shape of loading-unloading cycles.

Although the presented particle shape analysis is limited to only 25 stones per ballast type, it allows conclusions to be drawn regarding the experimental behaviour and DEM simulations of Calcite and Kieselkalk.

1. As no differences in the shape of Calcite and Kieselkalk stones were found, it is acceptable to conduct DEM simulations using the same shape for both materials, as it was done in^27^.
2. The different behaviours seen in Calcite and Kieselkalk in the uniaxial compression test, is most likely not caused by differences in shape between the two materials (as no difference could be found from the investigated stones).
3. To describe the observed differences in the uniaxial compression tests, focus should be laid on particle-particle contact modelling, taking into account differences in: friction (e.g. due to differences in roughness/texture as observed in this work), material behaviour (Young’s modulus, yield stress, etc.), etc. The combination of these material parameters will influence the material’s response both in compression and in shear. If both types of ballast differ in more than one material parameter, this is a possible explanation of the experimentally observed behaviour of differences in the uniaxial compression test, but similarity in the direct shear tests.

Related work addresses the particle shape modelling process in DEM simulations^36^, and future work further measurements of material properties of Calcite and Kieselkalk, e.g. Young’s modulus and coefficient of friction. All obtained data will be made openly available. This information will allow the development of new strategies for DEM model parametrisation and validation.

## Data Availability

The datasets generated and analysed during the current study are available in the zenodo.org repository^31^.

## References

[1] Jia X, Garboczi EJ (2016). Advances in shape measurement in the digital world. Particuology.

[2] Bagheri G, Bonadonna C, Manzella I, Vonlanthen P (2015). On the characterization of size and shape of irregular particles. Powder Technology.

[3] Blott SJ, Pye K (2008). Particle shape: a review and new methods of characterization and classification. Sedimentology.

[4] Wadell H (1932). Volume, shape, and roundness of rock particles. The Journal of Geology.

[5] Wadell H (1933). Sphericity and roundness of rock particles. The Journal of Geology.

[6] Wadell H (1935). Volume, shape, and roundness of quartz particles. The Journal of Geology.

[7] Bullard JW, Garboczi EJ (2013). Defining shape measures for 3d star-shaped particles: Sphericity, roundness, and dimensions. Powder Technology.

[8] Zhao B, Wang J (2016). 3d quantitative shape analysis on form, roundness, and compactness with $$\mu $$ CT. Powder Technology.

[9] Zhou B, Wang J, Wang H (2018). Three-dimensional sphericity, roundness and fractal dimension of sand particles. Geotechnique.

[10] Nie Z, Wang X, Liang Z, Gong J (2018). Quantitative analysis of the three-dimensional roundness of granular particles. Powder Technology.

[11] Nie Z, Liang Z, Wang X (2018). A three-dimensional particle roundness evaluation method. Granular Matter.

[12] Masad E, Saadeh S, Al-Rousan T, Garboczi E, Little D (2005). Computations of particle surface characteristics using optical and x-ray ct images. Computational Materials Science.

[13] Pan, T., Tutumluer, E. & Anochie-Boateng, J. Aggregate morphology affecting resilient behavior of unbound granular materials. In *Proceedings of the the 85th Annual Meeting of Transportation Research Board* (2006).

[14] Xiao J, Zhang D, Wei K, Luo Z (2017). Shakedown behaviors of railway ballast under cyclic loading. Construction and Building Materials.

[15] Al-Rousan T, Masad E, Tutumluer E, Pan T (2007). Evaluation of image analysis techniques for quantifying aggregate shape characteristics. Construction and Building Materials.

[16] Lee JRJ, Smith ML, Smith LN, Midha PS (2005). A mathematical morphology approach to image based 3d particle shape analysis. Machine Vision and Applications.

[17] Yang X, Chen S, You Z (2017). 3d voxel-based approach to quantify aggregate angularity and surface texture. Journal of Materials in Civil Engineering.

[18] Garboczi E, Liu X, Taylor M (2012). The 3-d shape of blasted and crushed rocks: From 20 $$\mu $$m to 38 mm. Powder Technology.

[19] Kong D, Fonseca J (2018). Quantification of the morphology of shelly carbonate sands using 3d images. Geotechnique.

[20] Le Pen M, Powrie W, Zervos A, Ahmed S, Aingaran S (2013). Dependence of shape on particle size for a crushed rock railway ballast. Granular Matter.

[21] Sun Y, Indraratna B, Nimbalkar S (2014). Three-dimensional characterisation of particle size and shape for ballast. Geotechnique Letters.

[22] Mollon G, Zhao J (2012). Fourier-Voronoi-based generation of realistic samples for discrete modelling of granular materials. Granular Matter.

[23] Latham, J.-P., Munjiza, A., Garcia, X., Xiang, J. & Guises, R. Three-dimensional particle shape acquisition and use of shape library for DEM and FEM/DEM simulation. *Minerals Engineering* **21**, 797–805 (2008). Discrete Element Methods (DEM).

[24] Ouhbi N, Voivret C, Perrin G, Roux J-N (2017). 3d particle shape modelling and optimization through proper orthogonal decomposition. Granular Matter.

[25] Anochie-Boateng JK, Komba JJ, Mvelase GM (2013). Three-dimensional laser scanning technique to quantify aggregate and ballast shape properties. Construction and Building Materials.

[26] Kozicki, J., Tejchman, J. & Mróz, Z. Effect of grain roughness on strength, volume changes, elastic and dissipated energies during quasi-static homogeneous triaxial compression using DEM. *Granular Matter* **14**, 457–468 (2012).

[27] Suhr B, Marschnig S, Six K (2018). Comparison of two different types of railway ballast in compression and direct shear tests: experimental results and DEM model validation. Granular Matter.

[28] Suhr, B. & Six, K. Compression tests and direct shear test of two types of railway ballast [data set]. 10.5281/zenodo.1423742 (2018).

[29] Harkness, J., Zervos, A. Le Pen, L., Aingaran, S. & Powrie, W. Discrete element simulation of railway ballast: modelling cell pressure effects in triaxial tests. *Granular Matter* **18**, 1–13 (2016).

[30] Suhr B, Six K (2017). Parametrisation of a DEM model for railway ballast under different load cases. Granular Matter.

[31] Suhr, B., Six, K., Skipper, W. A. & Lewis, R. 3D scans of two types of railway ballast including shape analysis information [dataset]. 10.5281/zenodo.3689592 (2020).

[32] Berghold A (2016). Wirkungsweise von unterschiedlichen Gleisschotterarten mit und ohne Schwellenbesohlung. ZEVrail.

[33] Cignoni, P. *et al*. MeshLab: an Open-Source Mesh Processing Tool. In *Eurographics Italian Chapter Conference* (2008).

[34] *PyMesh-Geometry Processing Library for Python* https://github.com/PyMesh/PyMesh (2018).

[35] Korsawe, J. Minimal bounding box http://www.mathworks.com/matlabcentral/fileexchange/18264-minimal-bounding-box (2008).

[36] Suhr, B. & Six, K. Simple particle shapes for DEM simulations of railway ballast: influence of shape descriptors on packing behaviour. *Granular Matter* **22**, 43, 10.1007/s10035-020-1009-0 (2020). 10.1007/s10035-020-1009-0 PMC7093080 32226281

